# Machine Learning‐Based Geospatial Risk Modeling of Global Avian Influenza Outbreaks

**DOI:** 10.1155/tbed/6615342

**Published:** 2026-04-20

**Authors:** Mehak Jindal, Samsung Lim, C. Raina MacIntyre

**Affiliations:** ^1^ School of Civil and Environmental Engineering, University of New South Wales, Sydney, 2052, New South Wales, Australia, unsw.edu.au; ^2^ Biosecurity Program, Kirby Institute, University of New South Wales, Sydney, 2052, New South Wales, Australia, unsw.edu.au; ^3^ College of Health Solutions and College of Public Service and Community Solutions, Arizona State University, Tempe, USA, asu.edu

**Keywords:** avian influenza, disease prediction, geospatial analysis, H5N1, machine learning, MaxEnt, risk mapping

## Abstract

The rapid spread of H5N1 avian influenza poses a global threat, highlighting the need for robust spatiotemporal risk assessment. In this study, we developed a global modeling framework integrating machine learning (ML) models and geospatial analysis to characterize H5N1 outbreak risk under varying environmental, ecological, and anthropogenic conditions. Confirmed H5N1 presence locations were extracted from World Animal Health Information System (WAHIS) (2012–2023), and pseudo‐absence locations were generated using a target‐group background (TGB) approach to account for heterogeneous surveillance effort. 5 ML algorithms, namely logistic regression (LR), support vector machines (SVMs), random forest (RF), light gradient boosting machine (LGBM), and extreme gradient boosting (XGB) were evaluated using spatial block cross‐validation on data from 2012 to 2021 and an independent temporal holdout dataset from 2022 to 2023. Tree‐based ensemble techniques (RF, LGBM, and XGB) achieved stronger and stable performance across both spatial and temporal validation. Seasonal Maximum Entropy (MaxEnt) models were applied to visualize broad‐scale outbreak risk patterns across the annual cycle. Seasonal maps revealed higher risk during autumn and winter, intermediate risk during spring migration, and reduced suitability during summer, consistent with large‐scale migratory connectivity, poultry production intensity, and seasonal environmental gradients. Predictor analysis indicated that livestock density and anthropogenic variables were the strongest correlates of outbreak occurrence in multivariate models, while wild bird abundance and climatic variables contributed heterogeneously and in a season‐dependent manner.

## 1. Introduction

Avian influenza is an infectious disease that was first identified in poultry in the early 1900s [1]. Over time, it has evolved into a zoonotic threat, with the first human infections being documented in 1997 [2]. Since 2020, there has been an unprecedented spread of the avian influenza virus (AIV) in both birds and mammals, driven largely by the H5N1 subtype of clade 2.3.4.4 b strain [3]. H5N1 has proven to be extremely infectious, lethal and has an expansive host range that now includes numerous wild bird species, various mammalian species, and humans. The mortality rate among infected humans is ~52% [4]. In 2005, the virus spread from Asia into Russia, Western Europe, Africa, and the Middle East, causing high mortality in wild bird populations [5]. By 2021, H5N1 had reached North America, with further spread to Central and South America in 2022. In the United States, the virus rapidly disseminated from the East Coast across the country, reaching the West Coast within ~4–6 months [6]. Infection of dairy cattle was documented for the first time in March 2024, and H5N1 now affects 19 States in the United States, including 758 outbreaks on dairy farms in California, the largest dairy producer in the country. Our research suggests that the first introduction into dairy farms in the United States was likely due to contact with wild birds [7]. H5N1 took about 10 months to spread along the western coast of South America, decimating populations of marine mammals such as sea lions. This suggests that the virus was introduced into the Pacific region from both the Asian and Atlantic flyways, indicating multiple independent incursions [8].

Past studies have examined the relationship between AIV outbreaks and socioeconomic and environmental factors. Migratory birds, particularly waterfowl, have been associated with the long‐distance spread of AIV along migration routes and are known as natural reservoirs of the AIV [9–12]. However, since 2020, H5N1 Infections have been documented across a much broader range of wild bird taxa, suggesting that outbreak risk may no longer be adequately described by traditional reservoir‐focused frameworks alone. In parallel, anthropogenic processes, poultry production systems, and environmental factors have been increasingly recognized as important contributors in AIV transmission [13–17].

Despite this growing complexity, global‐scale predictive modeling of H5N1 risk has remained limited, and many existing studies rely on region‐specific analyses or validation approaches that do not adequately account for spatial and temporal dependence. The rapid and uncontained spread of H5N1 underscores the urgent need to understand its transmission mechanisms. Machine learning (ML) methods offer the ability to integrate heterogeneous data sources and capture complex, nonlinear relationships, and help in identifying hidden patterns.

In this study, we develop a global modeling framework that integrates ML methods with geospatial analysis to characterize H5N1 outbreak risk under diverse environmental, ecological, and anthropogenic conditions. Confirmed outbreak locations were combined with pseudo‐absence data generated using a target‐group background (TGB) approach to better reflect heterogeneous surveillance effort. A diverse set of climatic variables, livestock density, wild bird abundance [18], anthropogenic proxies such as population density and nighttime light (NTL) intensity activity [19]. were assembled. Exploratory feature screening was employed to characterize predictor behavior and redundancy, while final model configuration was determined through spatial block cross‐validation and independent temporal hold‐out evaluation. By explicitly validating model performance across both space and time and by complementing ML predictions with seasonal presence‐only risk mapping, this study aims to provide a realistic and transferable assessment of global H5N1 risk patterns. The resulting framework is intended to support interpretation of seasonal outbreak dynamics and to inform surveillance strategies under evolving ecological and epidemiological conditions.

## 2. Materials and Methods

This section outlines the data sources, preprocessing steps, and modeling techniques used to assess the spatial and temporal risk distribution of H5N1 avian influenza outbreaks.

### 2.1. Data

#### 2.1.1. H5N1 Disease Records

World Animal Health Information System (WAHIS) is an open‐source database managed by the World Organization for Animal Health (WOAH) [20], 2025 that provides detailed reports on confirmed disease outbreaks with the latitude, longitude, outbreak location, and affected species. The spatial precision of reported locations may vary across countries and time periods. Some reports are likely reported at or near the outbreak site, while others may correspond to administrative centroids. WAHIS does not provide metadata indicating the spatial resolution of each record, preventing explicit stratification by locational accuracy. Confirmed H5N1 outbreak records spanning 2012–2023 were used as presence in this study. These records include outbreaks reported in poultry, wild birds and mammals. For wild bird outbreaks, species‐level information was compiled and aggregated into 11 avian taxonomic families to enable consistent ecological analysis at a global scale: Anseriformes (waterfowl such as ducks, geese, and swans), Accipitriformes (hawks and eagles), Charadriiformes (shorebirds and gulls), Ciconiiformes (herons and storks), Columbiformes (pigeons and doves), Galliformes (turkeys), Gruiformes (cranes and rails), Passeriformes (sparrows and finches), Pelecaniformes (pelicans and cormorants), Podicipediformes (grebes), and Strigiformes (owls).

#### 2.1.2. Bioclimatic Data

Giovanni is an Earth data portal that provides tools for visualizing, analyzing, and accessing remote sensing data. This study utilizes data from the Global Land Data Assimilation System (GLDAS) Noah Land Surface Model L4 Monthly 1.0° × 1.0° Version 2.1 (GLDAS_NOAH10_M) [21]. The dataset spans from January 2000 to December 2024, with a spatial resolution of 1.0° and a temporal resolution of 1 month [22, 23]. Each data file is ~2 MB in size and covers global latitudinal bounds from −60° to 90° and longitudinal bounds from −180° to 180°. GLDAS‐2.1 is reliable and has a large temporal and spatial coverage making it suitable for environmental modeling and geospatial analyses. The following environmental parameters were considered for modeling: air temperature, precipitation, soil moisture, specific humidity, surface air pressure, and wind speed.

##### 2.1.2.1. Geographic Landscape

Vegetation indices were obtained from the moderate resolution imaging spectroradiometer (MODIS) dataset to capture vegetation dynamics. Additionally, elevation data were obtained from the ASTER global digital elevation map (GDEM) [24]. Each pixel in the raster data represents a spatial resolution of 1 arc‐second, that is, an area of ~30 m × 30 m on the ground. The dataset provides global elevation coverage between 83°N and 83°S latitude. The elevation data for the year 2019 were used consistently across all years from 2005 to 2023 to ensure uniformity in the analysis.

#### 2.1.3. Bird Abundance Data

Weekly estimated wild bird abundance data were retrieved from eBird Status and Trends [25] for every affected wild bird species. Abundance estimates are produced at an approximate spatial resolution of 14 km × 14 km and represent modeled relative abundance rather than raw counts. Species‐level abundance estimates were retrieved for all wild bird species reported as affected by H5N1 in the WAHIS database. Each species was assigned to 1 of 11 avian taxonomic families: Accipitriformes, Anseriformes, Charadriiformes, Ciconiiformes, Columbiformes, Galliformes, Gruiformes, Passeriformes, Pelecaniformes, Podicipediformes, and Strigiformes. Family level aggregation reduces sparsity associated with species‐level reporting and supports integration with other predictors that vary at broader spatial resolutions. Weekly abundance estimates were aggregated to monthly averages to match the temporal resolution of other environmental predictors and outbreak records used in the modeling framework.

#### 2.1.4. Anthropogenic Data

##### 2.1.4.1. Population Density

The Gridded Population of the World dataset [26] was utilized to estimate human population density, indicating the number of people per square kilometer. It used a proportion‐assigning algorithm to assign the people count to 30‐arc second grids. The population density was then calculated by dividing the count by the land area. These data have been made available at 5‐year intervals (2000, 2005, 2010, 2015, and 2020). For this study, the most recent available dataset (2020) was used as a static proxy to represent human population distribution.

##### 2.1.4.2. Livestock Density

The Gridded Livestock of the World dataset was used to represent poultry species densities, including cattle, buffaloes, horses, sheep, goats, pigs, chickens, and ducks. This dataset has a 5‐min arc resolution (~0.083 decimal degrees) and provides data for 2015. Dasymetric weighed layers that represent livestock numbers disaggregated within census polygons were used. They are basically livestock numbers disaggregated within census polygons according to weights established by statistical models using high‐resolution spatial covariates [27]. Livestock density variables were treated as static representations of production intensity and spatial distribution rather than as temporally dynamic predictors.

##### 2.1.4.3. NTL

Anthropogenic activity and infrastructure were represented using NTL products derived from the visible infrared imaging radiometer suite (VIIRS) onboard the Suomi National Polar‐orbiting Partnership (Suomi NPP) satellite [28]. The VIIRS instrument includes the day/night band (DNB), which is specifically designed to detect low‐light emissions such as city lights and gas flares, enabling consistent observation of nocturnal human activity on a global scale. These data products are made freely available by the earth observation group (EOG) at the Payne Institute for Public Policy, Colorado School of Mines, in collaboration with the U.S. National Oceanic and Atmospheric Administration (NOAA). The VIIRS‐DNB NTL data is available from April 2012 onward [29] and has a spatial resolution of 15 arc‐seconds (~500 m) across a 3040 km swath, offering monthly global coverage. For this study, we utilized monthly averaged NTL composites, which were resampled to match the resolution and extent of other environmental and anthropogenic datasets used in the analysis [30].

### 2.2. Methods

A step‐by‐step approach was implemented to assess the environmental suitability and the outbreak risk of H5N1. First, a harmonized global dataset was assembled by integrating climatic, environmental, anthropogenic, and biological predictors from multiple sources. Second, a feature screening workflow was applied, combining univariate, multivariate, and model‐based selection techniques alongside multicollinearity diagnostics. Then candidate feature sets were used as inputs to 5 ML algorithms, including random forest (RF), support vector machines (SVMs), logistic regression (LR), light gradient boosting machine (LGBM), and extreme gradient boosting (XGB). Model performance was evaluated using the area under the receiver operating characteristic curve (AUC) and other performance metrics. Finally, seasonal Maximum Entropy (MaxEnt) models were used to generate spatial risk maps highlighting regions of high outbreak probability.

#### 2.2.1. Spatial Harmonization of Predictors

To ensure spatial consistency across datasets, all predictors were harmonized to a global grid of 1.0° × 1.0° in geographic coordinates (EPSG:4326). The GLDAS NOAH land‐surface variables (air temperature, precipitation, soil moisture, specific humidity, surface air pressure, and wind speed) are natively provided at this spatial resolution.

Predictors available at finer resolutions, including MODIS NDVI (250–500 m), ASTER GDEM elevation (30 m), GPWv4 population density (1 km), Gridded Livestock of the World poultry densities (0.083°), VIIRS‐DNB NTLs (500 m), and eBird abundance (~14 km^2^), were aggregated to the 1.0° grid using bilinear interpolation. This resolution was considered because all core climatic are available at this resolution and to ensure spatial alignment and avoiding upscaling errors. The aim of this study was to model broad‐scale risk patterns rather than local farm‐scale variability. Aggregating predictors to 1.0° reduces noise from fine‐scale fluctuations while retaining global environmental gradients and ensuring computational feasibility.

#### 2.2.2. Presence–Background Data Construction

WAHIS outbreak records provide presence‐only information. However, supervised ML algorithms require both presence (1) and absence (0) observations. To address this, absence points were considered using a TGB approach to account for spatial and reporting bias.

TGB sampling aims to approximate the spatial distribution of surveillance effort by drawing background points from locations where similar reporting processes occur, rather than from the entire geographic domain. This approach reduces bias arising from uneven reporting intensity and has been widely recommended for species distribution and disease risk modeling [31]. Background points were generated in Python by sampling locations from the same spatiotemporal domain as the observed outbreaks. This ensured that both presence and background points reflected comparable observation effort and data availability, avoiding unrealistic contrasts between well‐surveyed and poorly surveyed regions.

For each presence and background point, environmental and anthropogenic predictors were extracted using the point sampling tool in QGIS [32] from temporally matched raster datasets based on the outbreak date or sampling month. This presence‐background dataset formed the basis for all subsequent ML and MaxEnt analyses.

#### 2.2.3. Preprocessing and Exploratory Variable Screening

To prepare predictors for ML analysis, a structured preprocessing and exploratory screening pipeline was implemented to assess data quality, redundancy, and stability. This analysis was used to characterize predictor behavior and to inform the construction of alternative feature sets.

1. *Data ceaning and outlier detection*: Records containing missing values (NaN) were removed to ensure data consistency. Potential outliers were removed using the Isolation Forest algorithm [33]. This method is effective for high‐dimensional datasets since it does not assume any specific data distribution. Contamination rate is specified as a parameter during model training and defines the proportion of the most extreme data points to be flagged as anomalies. The anomaly score distribution was inspected, and approximately the lower tail of the distribution (~5% of observations) were excluded to reduce the influence of extreme outliers [34].
2. *Univariate analysis of variance* (*ANOVA*): Initial feature relevance was assessed using ANOVA [35], which quantifies the degree to which each predictor individually discriminates between presence and absence classes. Features were ranked using the ANOVA *F*‐statistics calculated using the Equation (1)

(1) F=Variance between groupsVariance within groups.

where groups are absence (0) and presence (1). High *F*‐value means the feature causes large separation between groups and is a strong predictor. Low *F*‐value means the differences in the feature are mostly just noise in the groups and might not be helpful in predicting the outcome.

3. *Multivariate screening using AIC:* To further assess the predictor relevance, we employed the AIC [36] within a generalized linear model framework. AIC measures how well a given feature contributes to predicting the response variable by assessing the log‐likelihood of the model. Since we were working with a moderate dataset size, we opted not to use the Bayesian information criterion (BIC), which applies a stricter penalty for additional parameters. AIC values were used comparatively to assess how predictor groupings influenced explanatory power while accounting for model complexity.
4. *Feature importance using RF*: RF feature importance [37] was computed to explore nonlinear relationships and interactions among predictors. Feature importance scores show the relative contribution of each predictor to reducing classification impurity across the ensemble, and hence help identifying the dominant environmental, anthropogenic, and biological drivers.
5. *Correlation and multicollinearity checks:* Pairwise correlation and variance inflation factors (VIF) were examined to identify groups of highly correlated predictors [38]. Tree‐based models were allowed to handle correlated predictors, while stability and performance were evaluated during cross‐validation.

Based on the exploratory assessments described above, multiple candidate feature sets were constructed, reflecting different assumptions about predictor relevance and redundancy. The candidate feature sets were evaluated using ML models. Final predictor retention was guided by spatially cross‐validated model performance.

#### 2.2.4. ML Models

To evaluate the risk of H5N1 outbreaks 5 ML models were implemented: LR, SVM, RF, LGBM, and XGB [39–43]. These models were chosen to capture both linear and complex nonlinear relationships between predictors and outbreak occurrences. Model training, feature‐set comparison, and hyperparameter tuning were conducted using data from the training period (2012–2021). Hyperparameter tuning was done using spatial block cross‐validation to account for spatial autocorrelation. Parameter search ranges and optimal configurations are summarized in Table S1.

For spatial validation, model performance was quantified using median performance across spatially independent folds. AUC was measured which assesses the ability of a model to distinguish outbreak presences from pseudo‐absences across all probability thresholds Given class imbalance, precision‐recall AUC (PR‐AUC) was additionally used to emphasize model performance in correctly identifying outbreak locations. Model calibration was evaluated using the Brier score, which quantifies the accuracy of predicted probabilities, while overall classification accuracy was reported as a summary measure.

To assess temporal generalization, final models were retrained on the full training dataset (2012–2021) and evaluated on an independent temporal hold‐out dataset from 2022 to 2023. The hold‐out evaluation incorporated both outbreak presences and target‐group pseudo‐absences. For the temporal hold‐out, discrimination, calibration, and classification performance were summarized using AUC, PR‐AUC, Brier score, accuracy, precision, recall, F1‐score, and confusion matrices for the final selected model.

#### 2.2.5. MaxEnt Analysis

MaxEnt modeling, a presence‐only framework [44] was employed to generate seasonal spatial risk maps of H5N1 outbreaks.

Confirmed H5N1 outbreak locations from the training period (2012–2021) were used as presence inputs. To account for changes in disease occurrence influenced by bird migration and climate, the dataset was divided into four seasonal periods of the northern hemisphere:

Season 1: December–February (winter).

Season 2: March–May (spring).

Season 3: June–August (summer).

Season 4: September–November (autumn).

For each season, the monthly raster layers of the environmental variables including temperature, precipitation, wind speed, and air pressure, were averaged to create seasonal composites. These seasonal predictors were used to fit MaxEnt models with linear and quadratic feature classes, allowing for nonlinear ecological responses while maintaining model interpretability. Season‐specific MaxEnt models were trained using data from 2012 to 2021 and subsequently projected onto environmental conditions from 2022 to 2023 to assess the temporal generalization of spatial risk patterns. Model performance was evaluated using AUC and omission rate (OR) metrics. MaxEnt outputs were interpreted as continuous relative suitability surfaces rather than binary classifications.

#### 2.2.6. Data Visualization and Analysis

A combination of spatial and statistical visualization techniques was employed to interpret model outputs and to examine H5N1 outbreak risk patterns. These visualizations helped in summarizing model performance, exploring predictor‐response relationships, and communicating spatiotemporal risk patterns.

1. *Model performance comparison*: ML models were evaluated quantitatively using spatial block cross‐validation and an independent temporal hold‐out period. Model calibration was assessed using AUC, PR‐AUC, Brier score, and overall accuracy. Performance summaries were reported using median values across spatial folds and final metrics for the temporal hold‐out period.
2. *Variable influence and response patterns*: MaxEnt partial response curves were generated to assess the influence of individual environmental variables on outbreak probability while holding other variables constant. This helps in understanding potential drivers of outbreak risk and the relationship between disease prevalence and ecological factors.
3. *Geospatial risk mapping*: Seasonal risk maps were generated using MaxEnt to visualize and highlight high‐risk regions. These maps provided insights into the spatiotemporal spread of H5N1 and risk variation across the annual cycle. Seasonal maps corresponding to winter (December–February), spring (March–May), summer (June–August), and autumn (September–November) were produced to illustrate how relative risk patterns vary across the annual cycle.

## 3. Results and Discussion

In this section, we present the results of exploratory variable screening, model performance evaluation, and spatiotemporal risk mapping.

### 3.1. Exploratory Feature Screening

To evaluate the robustness and relevance of candidate predictors, we applied multiple complementary screening approaches, including anomaly detection, univariate ranking, multivariate diagnostics, ML‐based importance measures, and collinearity assessment. These analyses were used to characterize predictor behavior and redundancy.

**Figure 1:**
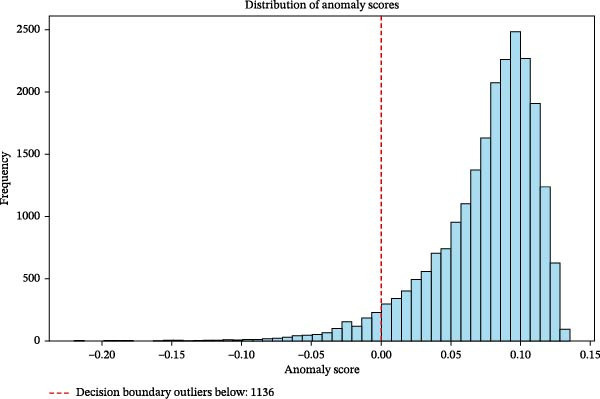
Distribution of anomaly scores using the isolation forest algorithm.

Figure 1 illustrates the distribution of anomaly scores derived using the Isolation Forest algorithm. The red dashed line represents the model’s decision boundary at an anomaly score of zero, which separates typical observations from anomalous patterns in the multivariate predictor space. Approximately 5% of observations in the lower tail of the anomaly score distribution were identified as extreme anomalies and excluded. The right‐skewed distribution indicates that only a small subset of observations deviated substantially from majority of the data.

**Figure 2:**
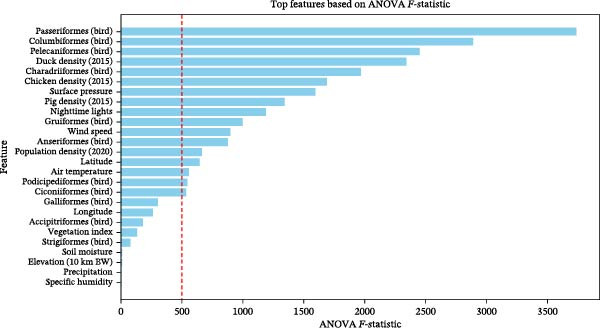
Top features using ANOVA scores.

Figure 2 displays the top features ranked by ANOVA *F*‐statistics, which quantify the degree of univariate separation between H5N1 outbreak presence and TGB pseudo‐absence locations. Bird abundance features and livestock density including Passeriformes, Columbiformes, Pelecaniformes, chicken, and cattle density were ranked among the highest, indicating strong univariate separation. Climatic and environmental variables generally ranked lower than bird abundance and livestock predictors. Air temperature showed moderate univariate separation, whereas precipitation, soil moisture, surface pressure, wind speed, and specific humidity exhibited comparatively low *F*‐statistics. Latitude and longitude ranked toward the lower end of the distribution. The ANOVA rankings were used to construct candidate predictor subsets of varying sizes.

To further examine predictor relevance in a multivariate context, stepwise regression using the AIC was conducted (Figure S1). The initial model exhibited a high AIC value, which decreased as predictors with negligible contributions to model likelihood were removed. Some predictors, including elevation and selected bird families like Accipitriformes, contributed minimally within this linear modeling framework.

**Figure 3:**
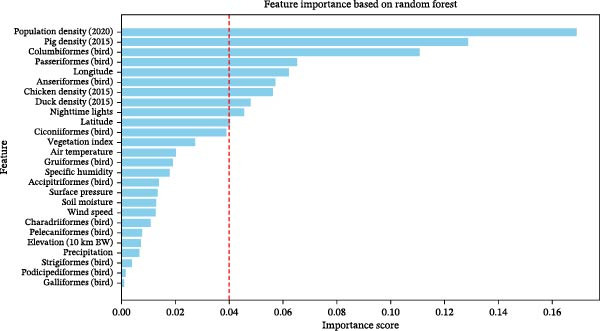
Top features using the random forest algorithm.

RF feature importance rankings are shown in Figure 3. The importance scores summarize how frequently and effectively each predictor contributed to reducing node impurity during tree construction and are used here to rank predictors according to their relative contribution in a nonlinear model. Livestock density variables and anthropogenic proxies, including cattle, chicken, pig density, and NTL, ranked highest, indicating that production intensity and human activity are dominant correlates of outbreak occurrence in the model. Wild bird abundance variables exhibited heterogeneous contributions. While Columbiformes and Passeriformes showed moderate importance, Anseriformes ranked lower relative to several other predictors, and no single bird group dominated the model. Climatic variables displayed comparatively lower importance scores in this ranking.

**Figure 4:**
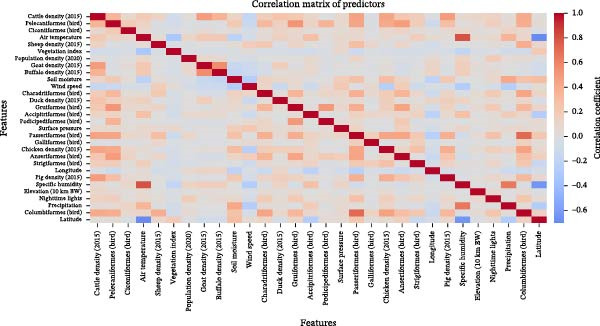
Correlation matrix between features.

Figure 4 displays the pairwise Pearsons correlation coefficients among environmental, anthropogenic, livestock, and wild bird abundance predictors. Overall, most predictor pairs exhibited weak to moderate correlations, indicating that no single variable was a near‐linear substitute for another across the global dataset.

Clusters of moderate correlation were observed among livestock density variables (cattle, chicken, pig, sheep, and buffalo densities), reflecting shared agricultural and production across regions. Similarly, several wild bird family abundance metrics showed moderate positive correlations, consistent with overlapping ecological niches and co‐occurrence patterns at broad spatial scales. Anthropogenic proxies such as population density and NTL were also moderately correlated with livestock densities, reflecting underlying links between human activity and animal production systems.

Climatic variables, including air temperature, precipitation, soil moisture, wind speed, specific humidity, and surface pressure, showed low‐to‐moderate pairwise correlations, with no single climatic variable showing uniformly strong correlation with all others. Latitude and longitude showed weak correlations with most predictors, reflecting large‐scale geographic gradients in climate and land use. Importantly, no pattern of near‐perfect multicollinearity was observed across the predictor set. While some variables formed correlated groups, particularly within thematic categories (e.g., livestock densities or bird families), correlations were not extreme.

**Figure 5:**
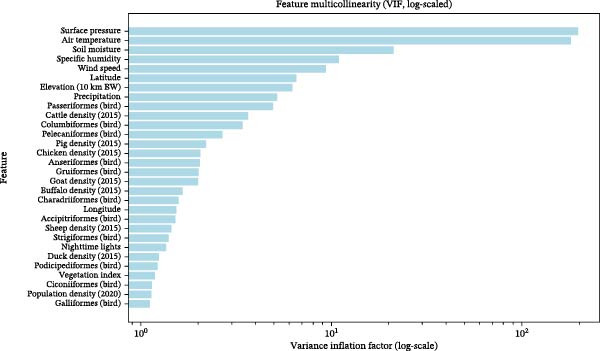
Variance inflation factor scores of features.

Figure 5 presents VIF values on a logarithmic scale to accommodate the wide range of multicollinearity levels observed across variables. Most climatic predictors exhibited high VIF values, reflecting a strong linear dependence among atmospheric variables. Moderate VIF values were observed for latitude and elevation, suggesting a partial association with climatic predictors. In contrast, most livestock density variables, wild bird family abundance metrics, vegetation index, and anthropogenic proxies (e.g., NTL and population density) exhibited relatively low VIF values, indicating limited linear redundancy.

Overall, exploratory feature screening revealed that no single predictor consistently dominated across univariate, multivariate, and nonlinear analyses and that outbreak risk is associated with a combination of livestock, anthropogenic, host‐related, and environmental factors. These analyses were therefore used to understand data structure, redundancy, and predictor behavior and to guide the construction and comparison of candidate feature sets. This was important for ensuring that subsequent ML models were evaluated within an informed framework, with final predictor configuration determined by spatially and temporally validated performance.

### 3.2. ML Model Performance

#### 3.2.1. Spatial Cross‐Validation Performance

Model performance under spatial block cross‐validation on training data from 2012 to 2021 is summarized in Table 1. Across all algorithms, high discriminatory performance was observed when evaluated on spatially independent folds, indicating strong generalization beyond localized outbreak clusters. Tree‐based models consistently outperformed linear approaches.

**Table 1:** Spatial block cross‐validation performance of machine‐learning models on training data (2012–2021).

Model	AUC median	PR AUC median	Brier median	Accuracy median
Logistic regression	0.948	0.987	0.088	0.905
Support vector machines	0.964	0.982	0.083	0.908
Random forest	0.984	0.996	0.065	0.929
Light gradient boosting machine	0.978	0.993	0.069	0.918
Extreme gradient boosting	0.983	0.996	0.079	0.903

RF achieved the highest median performance, with a median AUC of 0.984 (IQR: 0.957–0.990) and PR‐AUC of 0.996 (IQR: 0.965–0.998), accompanied by the lowest median Brier score (0.065). XGB and LGBM showed similarly strong discrimination, with median AUC values of 0.983 and 0.978, respectively. LR and SVM exhibited slightly lower but still high spatial performance, with median AUC values exceeding 0.94 across models. Overall, spatial cross‐validation results indicate that the models capture geographically transferable signal.

#### 3.2.2. Feature‐Set Sensitivity Under Spatial Validation

To evaluate sensitivity to predictor selection, multiple candidate feature sets informed by exploratory screening were compared under spatial block cross‐validation. Table S2 reports median performance metrics and ΔAUC values relative to the best‐performing feature set for each model. LR and SVM models showed greater sensitivity to feature reduction; however, their overall performance remained lower than that of tree‐based methods. Based on these results, the full predictor set was retained for final model training to maximize spatial generalization and stability. For all tree‐based models (RF, XGB, and LGBM), the full predictor set consistently achieved the highest spatially validated performance. Reduced feature sets including ecological and tree‐specific subsets resulted in low median AUC, often exceeding the predefined tolerance (ΔAUC ≤ 0.01). Feature sets informed by univariate ANOVA or RF rankings retained competitive performance in some cases but did not consistently match the full predictor set across models.

#### 3.2.3. Temporal Hold‐Out Evaluation

Final model performance was evaluated on an independent temporal hold‐out dataset from 2022 to 2023, incorporating both outbreak presences and pseudo‐absences (Table 2). Temporal performance closely mirrored spatial cross‐validation results, indicating robust generalization across time.

**Table 2:** Model performance on an independent temporal hold‐out dataset (2022–2023).

Model	Accuracy	Brier	Log loss	AUC	PR AUC
Random forest	0.948	0.038	0.133	0.990	0.977
Extreme gradient boosting	0.935	0.048	0.185	0.985	0.966
Light gradient boosting machine	0.932	0.053	0.196	0.981	0.959
Support vector machines	0.933	0.053	0.208	0.968	0.944
Logistic regression	0.946	0.046	0.227	0.952	0.941

RF achieved the strongest performance, with an AUC of 0.989, PR‐AUC of 0.977, and the lowest Brier score (0.038). XGB and LGBM also performed strongly, with AUC values of 0.984 and 0.981, respectively. LR and SVM exhibited lower but still high temporal discrimination, with AUC values exceeding 0.950.

Although ML models produced a strong predictive performance during model benchmarking, they rely on availability of presence‐absence data. In this study, the WAHIS database provides presence‐only outbreak reports. The pseudo‐absence points generated were suitable for comparative model evaluation but are not appropriate for global risk mapping as they may introduce spatial sampling bias. However, MaxEnt is based on presence‐only modeling and incorporates background sampling, regularization, and environmental constraints. Hence, MaxEnt is more robust for producing spatial risk maps for H5N1 and was adopted as the final modeling framework for generating risk projections, while the ensemble models were used to assess covariate importance and to benchmark predictive performance.

### 3.3. Seasonal MaxEnt‐Based Risk Mapping

Seasonal MaxEnt models were used as a presence‐only framework to visualize broad‐scale spatial patterns in H5N1 outbreak suitability across the annual cycle. Model performance metrics for each season are summarized in Table 3. Across all seasons, MaxEnt models achieved high discriminatory ability, with AUC values ranging from 0.90 to 0.93, indicating that outbreak locations were consistently differentiated from background locations in environmental space.

**Table 3:** Metrics for MaxEnt algorithm on presence data for different seasons.

Season	Omission rate	AUC
1 (December, January, February)	0.1235	0.9047
2 (March, April, May)	0.1510	0.9329
3 (June, July, August)	0.3435	0.9301
4 (September, October, November)	0.2653	0.9197

Season 2 (March–May) shows the highest AUC of (0.933), while Season 1 (December–February) had the lowest AUC of (0.905) as well as the lowest OR (0.123), indicating that a large proportion of outbreak locations were captured by the model during winter. The OR of Season 3 (June–August) is slightly higher (0.344) suggesting a reduced alignment between the selected environmental predictors and outbreak locations during the summer months. Seasonal differences in ORs highlight variation in how well presence‐only environmental suitability captures outbreak patterns across the year.

ROC curves for the four seasonal models (Figure S2) exhibit similar shapes, indicating stable discrimination across seasons. OR curves (Figure S3) show the expected trade‐off between sensitivity and specificity as suitability thresholds increase, with comparatively higher omission observed in summer, consistent with the seasonal AUC results.

**Figure 6:**
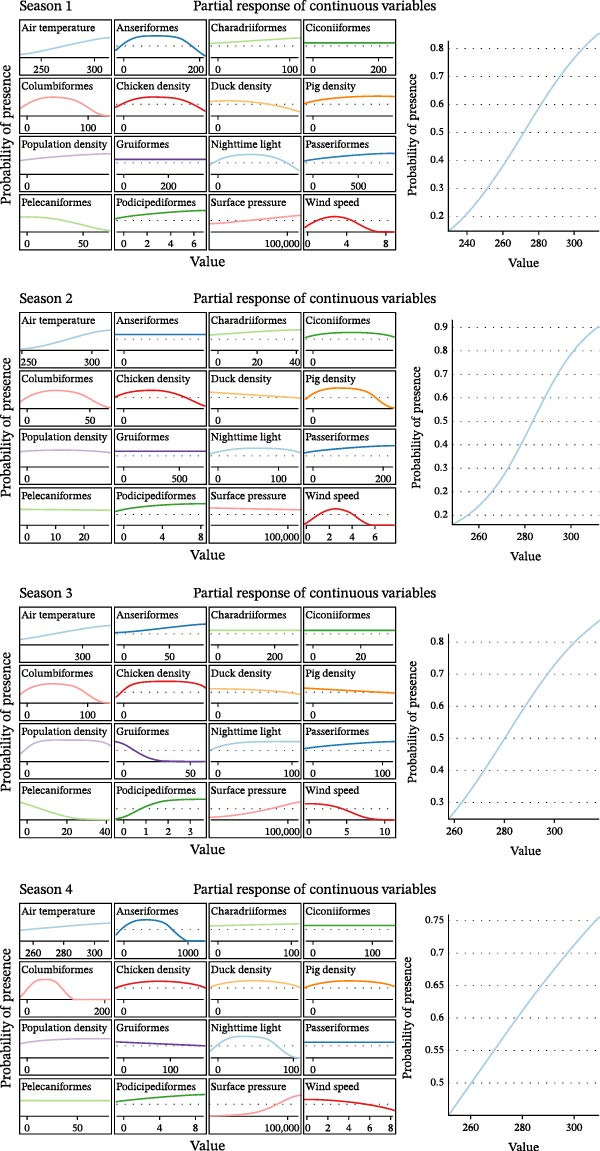
Partial response curve of every feature across different seasons using MaxEnt.

Partial response curves (Figure 6) illustrate how individual predictors influence predicted suitability within each seasonal model. Air temperature shows a positive association with predicted suitability across all seasons; however, this pattern likely reflects indirect associations with geographic, ecological, or anthropogenic gradients rather than a direct effect on viral survival. Livestock density variables (chicken, duck, and pig density) exhibit strong and generally monotonic associations with predicted suitability, highlighting the importance of poultry production systems in shaping outbreak risk patterns. Wild bird abundance variables show heterogeneous seasonal responses, with different bird families contributing variably across seasons. While Anseriformes, Columbiformes, and Podicipediformes display elevated suitability in some seasons, no single bird family consistently dominates across all seasonal models. Anthropogenic proxies, including population density and NTL, show positive associations with suitability in several seasons, indicating that human activity and production intensity are correlated with outbreak occurrence. Meteorological variables such as wind speed and surface pressure exhibit weaker and nonlinear responses, suggesting secondary or context‐dependent influence on suitability patterns.

Overall, the seasonal MaxEnt results highlight broad spatiotemporal patterns of relative outbreak suitability and seasonal variation in predictor‐response relationships. These maps are intended to support qualitative interpretation of seasonal risk dynamics and complement the spatially and temporally validated ML results. The observed seasonal variability underscores the importance of considering temporal context when interpreting environmental suitability for H5N1 outbreaks.

### 3.4. Seasonal Risk Mapping of H5N1 Outbreaks Using MaxEnt

**Figure 7:**
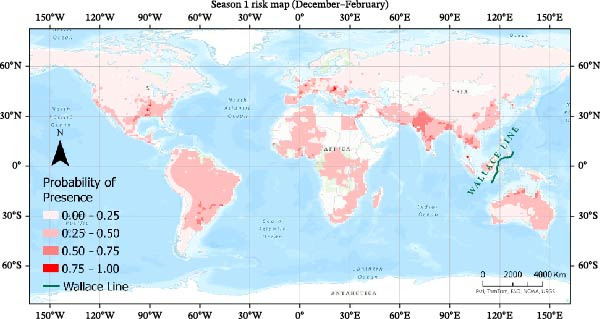
Risk map using MaxEnt for Season 1.

**Figure 8:**
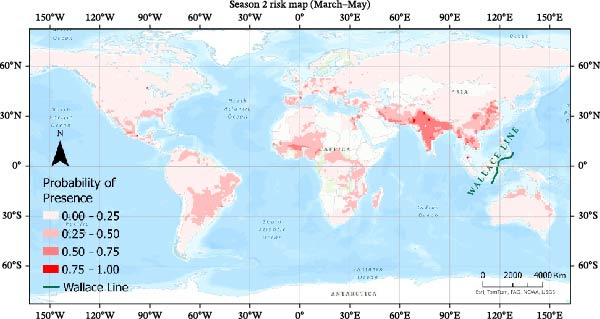
Risk map using MaxEnt for Season 2.

**Figure 9:**
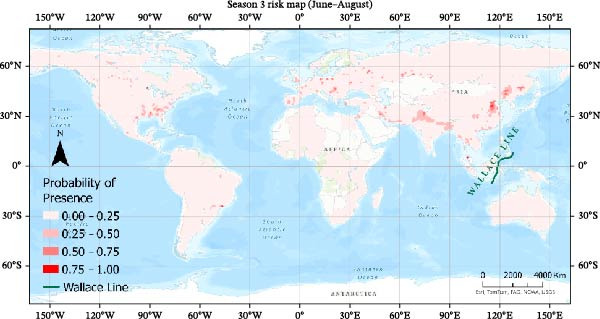
Risk map using MaxEnt for Season 3.

**Figure 10:**
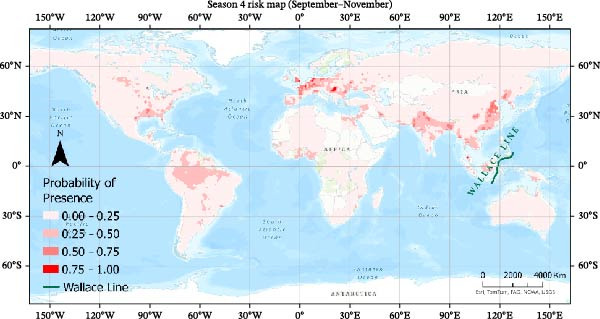
Risk map using MaxEnt for Season 4.

Seasonal MaxEnt models based on presence‐only outbreak data were used to map the relative probability of H5N1 occurrence across the annual cycle (Figures 7–10). Probabilities were grouped into four classes (0–0.25, 0.25−0.50, 0.50–0.75, 0.75−1.0) to highlight areas of elevated risk.

#### 3.4.1. Season 1 (December–February)

During the winter, extensive high‐probability zones are visible across Europe, the Eastern United States, and large parts of South and East Asia, particularly India and China (Figure 7). Elevated risk also appears in parts of South America (Argentina and Southern Brazil) and along coastal regions where migratory waterbirds overwinter and poultry production is dense. Northern and Eastern Australia show moderate suitability, but areas east of the Wallace Line remain largely at low risk, consistent with this biogeographical boundary that demarcates a stark difference in species composition between Southeast Asia and Australasia and may act as a natural barrier to the movement of certain bird species. This ecological natural barrier could help limit the southward spread of H5N1 to Australia by restricting the overlap of avian populations that usually carry the virus.

#### 3.4.2. Season 2 (March–May)

In spring, high‐risk regions persist across Europe and Asia, again with strong signals over the Indo‐Gangetic plain and Eastern China (Figure 8). This period coincides with northward migration toward breeding grounds, which likely concentrates infection risk along major flyways and stop‐over wetlands. Much of Africa shows low to moderate suitability, whereas risk in South America decreases relative to winter, reflecting shifting environmental suitability and migration routes.

#### 3.4.3. Season 3 (June–August)

Summer maps show a general reduction in predicted risk (Figure 9). Most regions display low to moderate probabilities, with only scattered hotspots remaining in parts of Northern Eurasia and localized areas of Asia. By this time, many migratory birds have reached breeding grounds, and long‐distance movement is reduced, which may lessen opportunities for long‐range spread. Residual risk during this period is likely driven more by local poultry production systems and resident bird populations than by large‐scale migration.

#### 3.4.4. Season 4 (September–November)

Autumn shows a renewed expansion of high‐probability areas, particularly across Europe and temperate Asia (Figure 10). A broad belt of elevated risk stretches from Western Europe through Central Asia into East Asia, mirroring southward return migration and use of agricultural and wetland stop‐over sites. India, Eastern China, and parts of the Mediterranean again emerge as persistent hotspots, consistent with large poultry populations and suitable environmental conditions. The spatial pattern in Season 4 closely resembles that of Season 1, indicating a cyclical risk structure driven by migration and seasonal climate.

Overall, the seasonal MaxEnt maps highlight a strong annual cycle in predicted H5N1 suitability: risk is highest during the main migration periods (Seasons 1 and 4), intermediate during spring migration (Season 2), and lowest when birds are largely settled on breeding or wintering grounds (Season 3). These patterns support the role of migratory connectivity and seasonal environments in shaping global H5N1 risk and underscore the value of season‐specific surveillance and control strategies, particularly along major flyways and in regions with dense poultry populations.

## 4. Conclusion

This study presents a global, spatiotemporally validated assessment of H5N1 outbreak risk using an integrated ML framework. By combining environmental conditions, livestock density, wild bird abundance, and anthropogenic proxies. Tree‐based ML models, particularly RF, consistently demonstrated the strongest and most stable performance under both spatial and temporal validation. Feature‐set sensitivity analyses showed that retaining the full predictor set yielded superior generalization compared with reduced subsets, indicating that outbreak risk is shaped by the combined influence of multiple correlated factors rather than a single dominant driver. Livestock density and anthropogenic variables emerged as the strongest correlates of outbreak occurrence in multivariate models, while wild bird abundance and climatic variables contributed heterogeneously and in a season‐dependent manner. Although certain wild bird families, including Columbiformes and Passeriformes, showed elevated associations with predicted risk in some analyses, no single avian group consistently dominated across models or seasons. Similarly, apparent positive associations with climatic variables such as temperature likely reflect indirect relationships with geographic, ecological, or anthropogenic gradients.

The presence‐only seasonal MaxEnt model was used to visualize broad‐scale patterns of relative outbreak suitability across the annual cycle. These maps revealed a clear seasonal rhythm, with elevated relative risk during autumn and winter, intermediate risk during spring migration, and reduced suitability during summer months. These patterns are consistent with large‐scale migratory connectivity, poultry production intensity, and seasonal environmental gradients. The risk maps suggest that Australia remains comparatively low risk relative to other continents, with limited suitability east of the Wallace Line. This biogeographical boundary may reduce overlap between Asian and Australasian avifauna and thus constrain long‐distance viral spread. However, the recent detection of H5N1 in Antarctica highlights the need for continued vigilance as changes in migratory connectivity or environmental conditions could alter regional risk profiles in the future.

Overall, this study demonstrates the value of integrating spatially and temporally validated ML models with presence‐only mapping to understand global H5N1 risk patterns. Future work could further refine predictions by incorporating dynamic poultry trade data, real‐time outbreak reporting, and explicitly spatiotemporal modeling approaches. Such advances would strengthen early warning systems and support more targeted surveillance and intervention strategies aimed at limiting the global spread of highly pathogenic avian influenza.

## Funding

No funding was received for this manuscript. Open access publishing facilitated by University of New South Wales, as part of the Wiley ‐ University of New South Wales agreement via the Council of Australasian University Librarians.

## Conflicts of Interest

The authors declare no conflicts of interest.

## Supporting Information

Additional supporting information can be found online in the Supporting Information section.

## Supporting information


**Supporting Information** Figure S1. AIC scores during stepwise regression. Figure S2. Receiver operating characteristic (ROC) curve for each season. Figure S3. Omission rate (OR) curve for each season. Table S1. Parameter search ranges and optimal configurations using spatial cross‐validation on training data (2012–2021). Table S2. Differences in median AUC (ΔAUC) across candidate feature sets for each machine‐learning model under spatial block cross‐validation (training period 2012–2021), relative to the best‐performing feature set for each model.

## Data Availability

Data available upon request from the authors.
