# Supplementary material for: Machine Learning‐Based Geospatial Risk Modeling of Global Avian Influenza Outbreaks
Source: Transbound Emerg Dis. 2026 Apr 20;2026:6615342. doi: 10.1155/tbed/6615342 (PMC13095849; doi:10.1155/tbed/6615342)
Supplement: Supplementary file 1 — Supporting Information Figure S1. AIC scores during stepwise regression. Figure S2. Receiver operating characteristic (ROC) curve for each season. Figure S3. Omission rate (OR) curve for each season. Table S1. Parameter search ranges and optimal configurations using spatial cross‐validation on training data (2012–2021). Table S2. Differences in median AUC (ΔAUC) across candidate feature sets for each machine‐learning model under spatial block cross‐validation (training period 2012–2021), relative to the best‐performing feature set for each model. [file TBED-2026-6615342-s001.docx]

**SUPPLEMENTARY MATERIAL**

**S1. AIC Scores during stepwise regression**


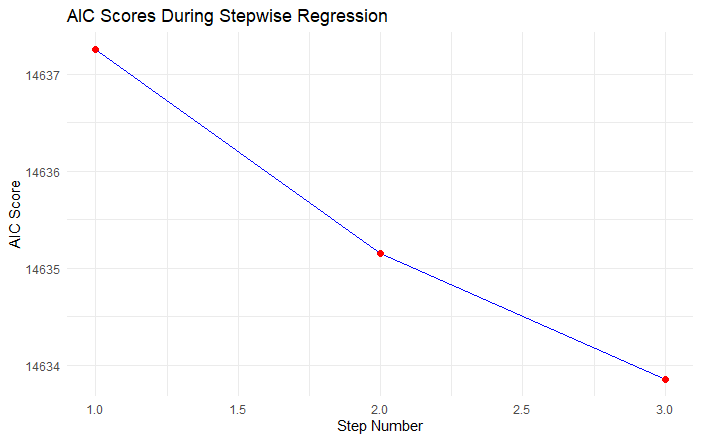


Figure S1: Stepwise regression AIC scores

**S2. Receiver Operating Characteristic (ROC) curve for each season**


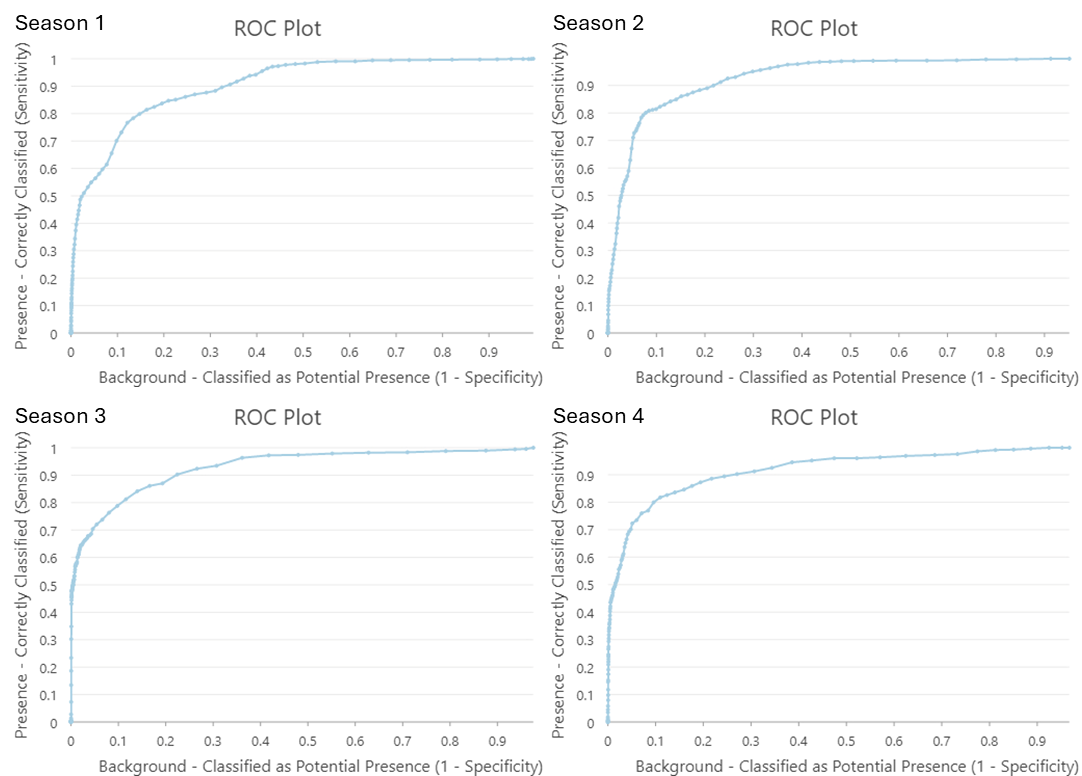


Figure S2: ROC plots across seasonal data using MaxEnt

**S3. Omission Rate (OR) curve for each season**


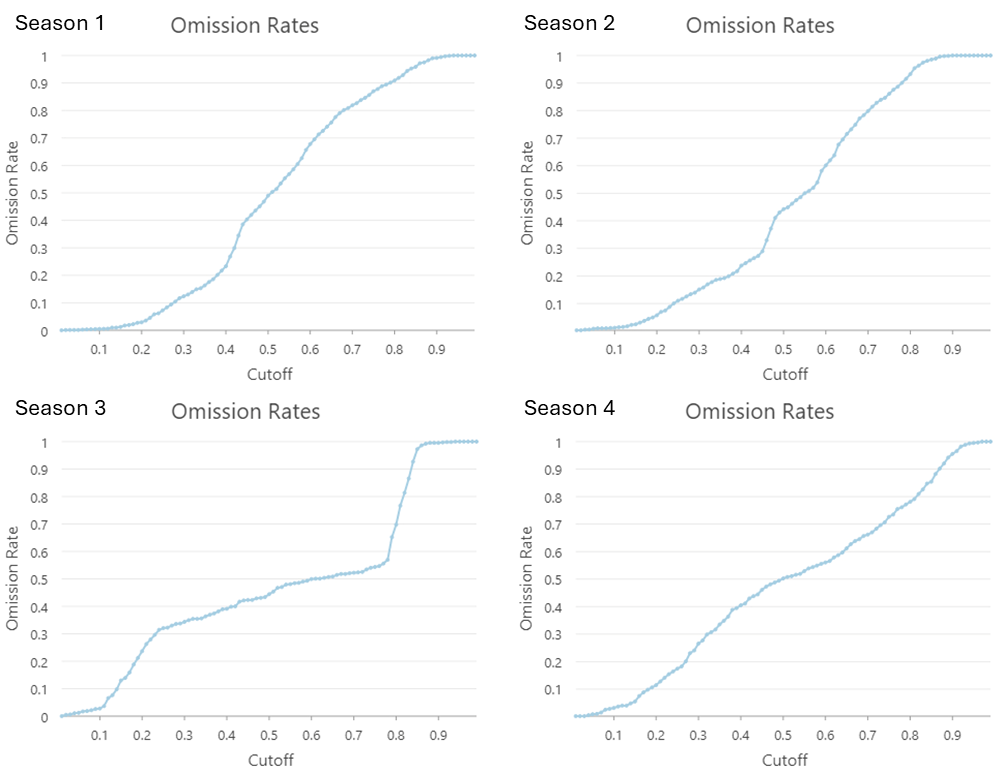


Figure S3: OR plots across seasonal data using MaxEnt

**S4. Parameter search ranges and optimal configurations using spatial cross-validation on training data (2012-2021)**

Table S1: Hyperparameter ranges and optimal values

| **Model** | **Hyperparameters Tuned** | **Search Range** | **Optimal Value(s)** |
| --- | --- | --- | --- |
| Logistic Regression  (LR) | Regularization (C) | [0.01, 0.1, 1, 10, 100] | 0.1 |
|  | Maximum iterations | [100,200,500] | 200 |
| Support Vector Machine (SVM) | Regularization (C) | [0.1, 1, 10, 100] | 10 |
|  | Kernel type | [linear, rbf] | rbf |
|  | Kernel coefficient (gamma) | [0.001, 0.01, 0.1, 1] | 0.01 |
| Random Forest  (RF) | Number of trees | [100, 300, 500] | 100 |
|  | Maximum depth | [5, 10, 20, None] | 10 |
|  | Minimum samples per leaf | [1, 5, 10] | 1 |
|  | Max features | [sqrt, log2, None] | sqrt |
| Light Gradient Boosting Machine (LGBM) | Learning rate | [0.01, 0.05, 0.1] | 0.05 |
|  | Number of estimators | [100, 300, 500] | 500 |
|  | Maximum depth | [3, 5, 7, 9] | 7 |
|  | Number of leaves | [21, 31, 61] | 31 |
| Extreme Gradient Boosting (XGB) | Learning rate | [0.01, 0.05, 0.1] | 0.1 |
|  | Number of estimators | [100, 300, 500] | 300 |
|  | Maximum depth | [4, 7, 10] | 4 |
|  | Subsample | [0.7, 0.9, 1.0] | 0.9 |

**S5. Differences in median AUC (ΔAUC) across candidate feature sets for each machine-learning model under spatial block cross-validation (training period 2012–2021), relative to the best-performing feature set for each model.**

Table S2: ΔAUC across candidate feature sets for each model

| **Model** | **Feature set** | **AUC**  median | Δ**AUC** | **Within AUC Tolerance?** |
| --- | --- | --- | --- | --- |
| Logistic | All features | 0.951 | -0.044 | FALSE |
| Logistic | Ecology | 0.851 | -0.144 | FALSE |
| Logistic | Linear | 0.938 | -0.057 | FALSE |
| Logistic | Overlap ANOVA & RF | 0.981 | -0.014 | FALSE |
| Logistic | Top ANOVA | 0.995 | 0.000 | TRUE |
| Logistic | Top RF | 0.980 | -0.016 | FALSE |
| Logistic | Tree | 0.938 | -0.058 | FALSE |
| SVM | All features | 0.964 | -0.022 | FALSE |
| SVM | Ecology | 0.858 | -0.128 | FALSE |
| SVM | Linear | 0.952 | -0.034 | FALSE |
| SVM | Overlap ANOVA & RF | 0.982 | -0.005 | TRUE |
| SVM | Top ANOVA | 0.972 | -0.014 | FALSE |
| SVM | Top RF | 0.987 | 0.000 | TRUE |
| SVM | Tree | 0.958 | -0.028 | FALSE |
| RF | All features | 0.987 | -0.003 | TRUE |
| RF | Ecology | 0.917 | -0.073 | FALSE |
| RF | Linear | 0.974 | -0.017 | FALSE |
| RF | Overlap ANOVA & RF | 0.990 | 0.000 | TRUE |
| RF | Top ANOVA | 0.990 | -0.001 | TRUE |
| RF | Top RF | 0.990 | 0.000 | TRUE |
| RF | Tree | 0.964 | -0.026 | FALSE |
| LGBM | All features | 0.989 | 0.000 | TRUE |
| LGBM | Ecology | 0.870 | -0.119 | FALSE |
| LGBM | Linear | 0.966 | -0.023 | FALSE |
| LGBM | Overlap ANOVA & RF | 0.979 | -0.010 | FALSE |
| LGBM | Top ANOVA | 0.980 | -0.009 | TRUE |
| LGBM | Top RF | 0.989 | 0.000 | TRUE |
| LGBM | Tree | 0.964 | -0.025 | FALSE |
| XGB | All features | 0.989 | -0.001 | TRUE |
| XGB | Ecology | 0.907 | -0.083 | FALSE |
| XGB | Linear | 0.972 | -0.018 | FALSE |
| XGB | Overlap ANOVA & RF | 0.989 | -0.001 | TRUE |
| XGB | Top ANOVA | 0.988 | -0.002 | TRUE |
| XGB | Top RF | 0.990 | 0.000 | TRUE |
| XGB | Tree | 0.959 | -0.031 | FALSE |
